# PI3Kδ and PI3Kγ isoforms have distinct functions in regulating pro-tumoural signalling in the multiple myeloma microenvironment

**DOI:** 10.1038/bcj.2017.16

**Published:** 2017-03-10

**Authors:** R E Piddock, N Loughran, C R Marlein, S D Robinson, D R Edwards, S Yu, G E Pillinger, Z Zhou, L Zaitseva, M J Auger, S A Rushworth, K M Bowles

**Affiliations:** 1 Department of Molecular Haematology, Norwich Medical School, The University of East Anglia, Norwich Research Park, Norwich, UK; 2 School of Biological Sciences, The University of East Anglia, Norwich Research Park, Norwich, UK; 3 Norwich Medical School, The University of East Anglia, Norwich Research Park, Norwich, UK; 4 Department of Haematology, Norfolk and Norwich University Hospitals NHS Trust, Colney Lane, Norwich, UK

## Abstract

Phosphoinositide-3-kinase and protein kinase B (PI3K-AKT) is upregulated in multiple myeloma (MM). Using a combination of short hairpin RNA (shRNA) lentivirus-mediated knockdown and pharmacologic isoform-specific inhibition we investigated the role of the PI3K p110γ (PI3Kγ) subunit in regulating MM proliferation and bone marrow microenvironment-induced MM interactions. We compared this with inhibition of the PI3K p110δ (PI3kδ) subunit and with combined PI3kδ/γ dual inhibition. We found that MM cell adhesion and migration were PI3Kγ-specific functions, with PI3kδ inhibition having no effect in MM adhesion or migration assays. At concentration of the dual PI3Kδ/γ inhibitor duvelisib, which can be achieved *in vivo* we saw a decrease in AKT phosphorylation at s473 after tumour activation by bone marrow stromal cells (BMSC) and interleukin-6. Moreover, after drug treatment of BMSC/tumour co-culture activation assays only dual PI3kδ/γ inhibition was able to induce MM apoptosis. shRNA lentiviral-mediated targeting of either PI3Kδ or PI3Kγ alone, or both in combination, increased survival of NSG mice xeno-transplanted with MM cells. Moreover, treatment with duvelisib reduced MM tumour burden *in vivo*. We report that PI3Kδ and PI3Kγ isoforms have distinct functions in MM and that combined PI3kδ/γ isoform inhibition has anti-MM activity. Here we provide a scientific rationale for trials of dual PI3kδ/γ inhibition in patients with MM.

## Introduction

Multiple Myeloma (MM), is presently incurable with <50% of patients surviving over 5 years post diagnosis.^1^ MM is characterised by the accumulation of monoclonal plasma cells that are primarily contained within the bone marrow. Current treatments including proteasome inhibitors, immune modulating drugs and alkylating agents are effective at reducing the tumour bulk and alleviating symptoms,^2, 3^ however relapse remains inevitable from residual disease sequestered within the MM microenvironment. It is envisaged that improved patient outcomes will come from novel therapeutic strategies informed by an improved understanding of the biology of the disease.^4^

Phosphoinositide-3-kinases (PI3K) are an enzyme group that generate phosphatidylinositol 3,4, 5-triphosphate (PIP_3_). PIP_3_ provides a membrane docking site for the tyrosine kinase AKT (also known as protein kinase B), which on binding upregulates cell survival and proliferation signals. Class 1 group of PI3K subunits in particular (PI3K α/β/γ/δ) are known to be involved in the carcinogenesis and chemo-resistance in several cancer types. PI3Kδ is usually activated by receptor tyrosine kinase signalling, however PI3Kγ is most commonly found downstream of G protein-coupled receptors.^5, 6, 7^ PI3K α/β catalytic subunits are expressed in a wide variety of tissues, whereas PI3Kδ/γ have been shown to be specifically enriched in the haematopoietic system.^8^

The aberrant activation of the PI3K-AKT pathway has been reported in several blood cancers including acute myeloid leukaemia, chronic lymphocytic leukaemia and MM.^9, 10^ Inhibition of pan-PI3K using LY295002- and PI3Kδ-only inhibition using idelalisib (previously known as CAL-101) has been shown to downregulate chronic lymphocytic leukaemia and MM cell survival signals,^11^ causing malignant cells to become more sensitive to chemotherapeutic treatment.^12, 13^ Moreover, idelalisib is currently licensed for the treatment of selected patients with chronic lymphocytic leukaemia and follicular lymphoma.^14^ Interestingly, PI3Kγ has been shown to be highly expressed in MM cells,^11^ however the exact function of PI3Kγ subunit in the biology of MM is not yet defined. Using drugs that inhibit PI3Kδ (idelalisib), PI3Kγ (CZC24832) or both PI3Kδ and PI3Kγ in combination (duvelisib; previously known as IPI-145) and experiments using short hairpin RNA (shRNA) knockdown, we investigated the pharmacological and molecular outcome of targeting PI3Kδ and PI3Kγ both alone and in combination in MM using *in vitro* and *in vivo* assays.

## Materials and methods

### Materials

Anti-phosphorylated and pan, AKT, and MAPK antibodies, and PI3Kα/β/γ antibodies were purchased from Cell Signalling Technology (Cambridge, MA, USA). Anti-PI3Kδ antibody was purchased from R&D systems (Oxford, UK). Anti-CD138-PE, anti-CD90-FITC, anti-CD73-PE, anti-CD105-APC antibodies (Cat. 130-098-122, 130095403, 130095182, 130094926) and interleukin-6 (IL-6) were purchased from Miltenyi Biotec (Auburn, CA, USA). Idelalisib, CZC24832, duvelisib were obtained from Selleck Chemicals (Houston, TX, USA). All other reagents were obtained from Sigma-Aldrich (St Louis, MO, USA), unless otherwise indicated.

### Cell lines and primary samples

The MM-derived cell lines were obtained from the European Collection of Cell Cultures where they are authenticated by DNA fingerprinting. MM cell lines were cultured in RPMI 1640 medium supplemented with 10% foetal bovine serum, penicillin and streptomycin (all obtained from Invitrogen, Paisley, UK). Primary MM cells were obtained from patients' bone marrow after informed consent was given in accordance with the Declaration of Helsinki and under approval from the United Kingdom National Research Ethics Service (07/H0310/146).

For primary cell isolation, heparinised bone marrow was collected from volunteers; human bone marrow cells were isolated by histopaque density-gradient centrifugation and plated in growth media. Non-adherent cells were removed after 24 h. At 60–80% confluency, adherent cells were trypsinised and expanded for 3–6 weeks. bone marrow stromal cells (BMSCs) were checked for positive expression of CD105, CD73 and CD90, and the lack of expression of CD45 by flow cytometry as previously described.^15^ Primary plasma cells were purified by positive selection using magnetic-activated cell sorting with CD138^+^ MicroBeads (Miltenyi Biotec).

### Viability and apoptosis assay

Cell lines were plated in quintuplicate in 96-well flat-bottom plates with idelalisib, CZC24832 and duvelisib. These were incubated for 24–72 h with viable numbers being measured using Cell Titre GLO (Promega, Southampton, UK). Flow cytometry was performed to measure apoptosis using the CyFlow Cube 6 flow cytomter (Sysmex, Milton Keynes, UK). For measuring viability, samples were collected and stained with Annexin V and propidium iodide (PI), followed by detection via flow cytometry. Data were then normalised to vehicle controls. All data points are represented as the mean with s.d.

### Western immunoblotting

SDS–polyacrylamide gel electrophoresis and Western analyses were performed as described previously.^16^ Briefly, whole-cell lysates were extracted using radio immunoprecipitation assay buffer method and SDS–polyacrylamide gel electrophoresis separation was performed. Protein was transferred to PVDF membrane and Western blot analysis performed with the indicated anti-sera according to manufacturer's guidelines. Detection was performed by electrochemical luminescence.

### BMSC/fibronectin–MM cell adhesion assay

BMSCs were grown in 96-well tissue culture plates at 2 × 10^4^ cells per well in 200 μl of media. MM cells were incubated with 2.5 μm calcein AM for 1 h at 37°C and 5% CO_2_. The fluorescence-labelled MM cells were added to BMSC plates and incubated for the indicated time points. Non-adherent calcein-labelled cells were removed by gently washing and adherent cells were quantitated in a fluorescence multi-well plate reader. For MM cell adhesion onto fibronectin (FN), 96-well plates were coated with 10 mg/ml FN for 1 h before the fluorescence-labelled MM cells were added. Non-adherent calcein-labelled cells were removed by gently washing and adherent cells were quantitated in a fluorescence multi-well plate reader.

### Lentiviral transduction

pCDH-luciferase-T2A-mCherry was kindly gifted from Professor Dr med Irmela Jeremias, Helmholtz Zentrum München, Munchen, Germany.^17^ Lentivirus particles generated using this construct were produced as previously described.^18^ Lentiviral stocks were concentrated using Amicon Ultra centrifugal filters and titres were determined using Lenti-X qRT-PCR titration kit (CloneTech, Oxford, UK). U266 cells were plated at a density of 5 × 10^4^ per well in a 12-well plate and expanded. U226 cells expressing mCherry (U266-luc) were sorted on a FACSAria (BD, Oxford, UK).

### Migration assays

Migration assays were performed in triplicate in transwell permeable plates with 4.0 μm pores (Neuroprobe, Gaithersburg, MD, USA). The lower compartment contained 30 μl of conditioned media or serum-free media supplemented with 100 ng/ml stromal cell derived factor 1 (SDF1). Myeloma cells were applied to the upper compartment and allowed to migrate for 4 h. The quantity of viable migrated myeloma cells was determined by counting using trypan blue exclusion and expressed as a percentage of the input.

### Real-time PCR

Total RNA was extracted from cells using the ReliaPrep RNA extraction kit from Promega according to the manufacturer's instructions. Reverse transcription was performed using the qPCRBIO cDNA synthesis kit (PCR Biosystems, London, UK). Relative quantitative real-time PCR using qPCRBIO SyGreen Mix (PCR Biosystems) was performed on complimentary DNA generated from the reverse transcription of purified RNA. After pre-amplification (95°C for 2 min), the PCRs were amplified for 45 cycles (95°C for 15 s, 60°C for 10 s and 72°C for 10 s) on a 384-well LightCycler 480 (Roche, Burgess Hill, UK). Each messenger RNA expression was normalised against β-actin messenger RNA expression.

### MM xenograft model

For this study NOD.Cg-Prkdcscid IL2rgtm1Wjl/SzJ (NSG) mice from The Jackson Laboratory, Bar Harbour, ME, USA were used. The NSG mice were maintained under specific pathogen-free conditions. All animal experiments were performed in accordance with UK Home Office regulations. For the MM xenograft model, 0.5 × 10^6^ U266-luc cells were intravenously injected into non-irradiated 6–8-week-old NSG mice. On day 4 after intravenously injection of U266 cells, NSG mice were treated with duvelisib at 15 mg/kg per day via intraperitoneal injection. Mice were treated for 10 days with duvelisib (*n*=6) or vehicle control (*n*=6).

For lentiviral experiments, either control knockdown (Con KD) or target gene KD luciferase expressing U266 cells were intravenously injected into non-irradiated 6–8-week-old NSG mice. When clinical signs of illness became apparent mice were killed by exposure to CO_2._ Bone marrow was harvested and analysed for human CD45. All mice were monitored via *in vivo* bioluminescent imaging (Bruker, Coventry, UK).

### Statistical analysis

For Western blotting experiments, data are representative of three independent experiments. For all *in vitro* assays, a Mann–Whitney *U*-test was performed to assess statistical significance against the control group. The Mantel–Cox test was used to analyse Kaplan–Meier survival curves.

## Results

### Inhibition of PI3K PI3Kδ and PI3Kγ subunits reduces MM proliferation and survival

**Figure 1:**
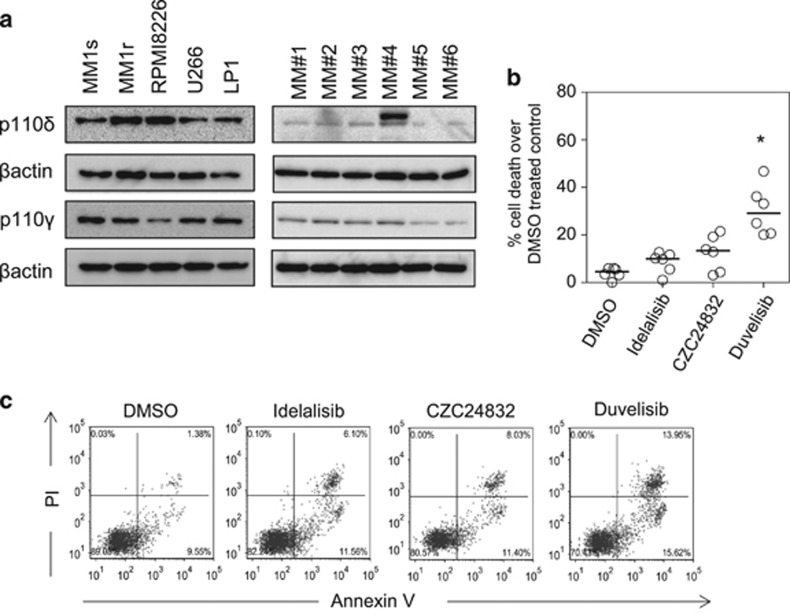
Inhibition of PI3Kδ and PI3Kγ subunits in MM proliferation. (**a**) Western blot analysis of PI3Kδ and PI3Kγ subunits in MM cell lines and MM primary cells. Blots were probed with β-actin to show sample loading. (**b**) MM primary cells were treated with idelalisib (1 μm), CZC24832 (1 μm) and duvelisib (1 μm) for 72 h and then assessed for cell viability using CellTiter Glo. (**c**) MM primary cells were treated with idelalisib (1 μm), CZC24832 (1 μm) and duvelisib (1 μm) for 72 h and then assessed for apoptosis by PI/Annexin V staining (*n*=4).

To verify that PI3Kδ and PI3Kγ subunits are expressed in MM cells we examined protein expression of both isoforms using Western blotting in primary MM samples and MM cell lines. Figure 1a shows that both isoforms are expressed in MM cell lines and primary MM cells. To understand the significance of PI3Kδ and/or PI3Kγ in MM we investigated the effects of PI3K inhibitors (idelalisib (PI3Kδ), CZC24832 (PI3Kγ) and duvelisib (PI3Kδ/γ)) on MM cell death assays. As idelalisib and duvelisib are active at nanomolar concentrations^19, 20^ and low micromolar concentrations are achievable *in vivo*,^21, 22^ we performed *in vitro* experiments using idelalisib (1 μm), CZC24832 (1 μm) and duvelisib (1 μm). Drug was added to assays for 72 h and MM cell death was measured using the CellTiter Glo assay. At the concentration used the MM primary cells have increased cell death after dosing with duvelisib (*P*<0.01; Figure 1b). Furthermore, we show that duvelisib had a greater effect on primary MM apoptosis than either idelalisib or CZC24832 (Figure 1c).

### Combined knockdown of PI3Kδ/γ isoforms reduces survival and increases apoptosis MM cell lines

**Figure 2:**
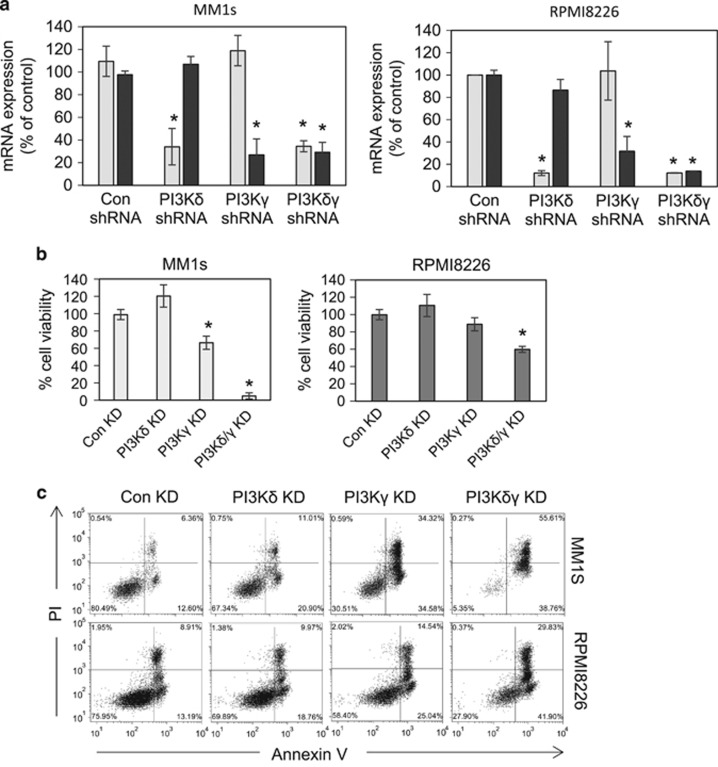
Knockdown of PI3K isoforms in MM. (**a**) MM1s cells and RPMI8226 were transduced with lentivirus targeted to PI3Kδ and PI3Kγ or control shRNA for 72 h. RNA was extracted and analysed for either PI3Kδ or PI3Kγ messenger RNA expression by real-time PCR. GAPDH was used to normalise RNA values. (**b**) MM1s cells and RPMI8226 were transduced with lentivirus targeted to PI3Kδ and PI3Kγ or control shRNA for 5 days. Cells were then assayed for viability by Cell Titre Glo assay. (**c**) MM1s cells and RPMI8226 were transduced with lentivirus targeted to PI3Kδ and PI3Kγ or control shRNA for 5 days. Cells were then assessed for apoptosis by PI/Annexin V staining.

To help determine the dominant PI3K isoform in MM proliferation and downstream signalling we used targeted shRNA KD of PI3Kδ and PI3Kγ messenger RNA. Results confirm that the PI3Kδ and PI3Kγ shRNA caused significant KD of PI3Kδ and PI3Kγ messenger RNA in MM1s and RPMI8226 cells (Figure 2a). Figure 2b shows that PI3Kδ had no effect on MM1s or RPMI8226 cell line viability, however PI3Kγ KD had a significant effect on MM1s but not RPMI8226 cells. Furthermore, combined knockdown of PI3Kδ and PI3Kγ had a significant effect in reducing *in vitro* survival of both MM1s and RPMI8226 cells. Figure 2c shows that combined knockdown of PI3Kδ and PI3Kγ also increased apoptosis in both MM1s and RPMI8226 cells.

### Combined KD of PI3Kδ and PI3Kγ is more effective at inhibiting AKT phosphorylation than inhibition of either isoform alone

**Figure 3:**
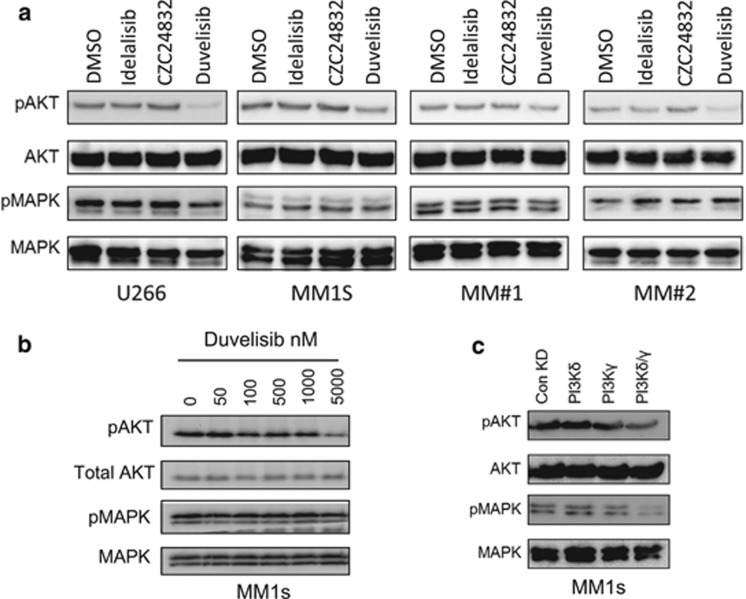
Inhibition of PI3Kδ and PI3Kγ inhibits AKT phosphorylation. (**a**) MM1s, U266, MM#1 and MM#2 cells were incubated with idelalisib (1 μm), CZC24832 (1 μm) and duvelisib (1 μm) for 4 h after which protein was extracted. Samples were subsequently analysed for phospho-AKT and phospho-MAPK responses using Western blotting. Blots were re-probed for total AKT and MAPK to confirm sample loading. (**b**) MM1s were incubated with increasing doses of duvelisib for 4 h after which protein was extracted. Samples were then analysed for phospho-AKT and phospho-MAPK response using Western blotting. Blots were re-probed for total AKT and MAPK to confirm sample loading. (**c**) MM1s cells were transduced with lentivirus targeted to PI3Kδ and PI3Kγ or control shRNA for 72 h. Protein was extracted and analysed for phospho-AKT and phospho-MAPK via Western blotting. Total AKT and MAPK were used as loading controls.

As PI3Kδ and PI3Kγ are known to activate AKT, we analysed the phosphorylation status of s473 in response to idelalisib, CZC24832 and duvelisib. Drugs were used at a dose of 1 μm. Figure 3a shows that duvelisib inhibits the constitutive phosphorylation of s473 in MM1S cells, U266 cells and primary MM samples. However, idelalisib (P3Kδ) or CZC24832 (PI3Kγ) had no inhibitory effect on AKT phosphorylation on U266, MM1s and primary MM cells at the drug concentration used. In addition, no inhibition of MAPK phosphorylation was observed with any of the drugs. Figure 3b shows that in MM1s cells duvelisib inhibits constitutive phosphorylation of AKT at s473 in a dose-dependent manner. Next we examined if knockdown of either PI3Kδ or PI3Kγ could inhibit AKT phosphorylation. Figure 3c shows that combined KD of PI3Kδ and PI3Kγ was more effective at inhibiting AKT phosphorylation than knockdown of either isoform alone. These results demonstrate that inhibiting both PI3Kδ and PI3Kγ using duvelisib or by genetic knockdown has a greater effect on AKT s473 inhibition than PI3Kδ or PI3Kγ inhibition alone.

### MM adhesion to BMSC is regulated by both PI3Kδ and PI3Kγ, with PI3Kγ exclusively mediating fibronectin adhesion and MM cell migration

**Figure 4:**
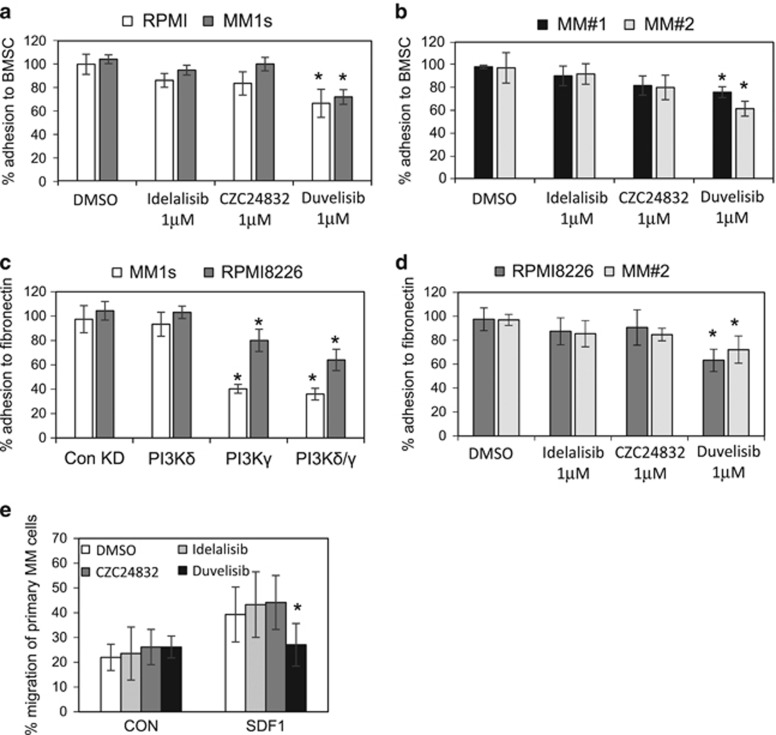
MM adhesion to BMSC and fibronectin is regulated by PI3Kδ. (**a**, **b**) MM cell lines and MM primary cells were treated with idelalisib (1 μm), CZC24832 (1 μm) and duvelisib (1 μm) for 4 h and then stained with calcein AM. Stained cells were added to BMSC on 96-well plates for a 4 h incubation. Non-adherent cells were removed and fluorescence was measured. (**c**) MM1s and RPMI8226 cells were transduced with lentivirus targeted to PI3Kδ and PI3Kγ or control shRNA for 72 h. Transduced cells were stained with calcein AM and added to BMSC on a 96-well plate for a 4 h incubation. Non-adherent cells were removed and fluorescence was measured. (**d**) RPMI8226 and MM#2 primary cells were treated with idelalisib (1 μm), CZC24832 (1 μm) and duvelisib (1 μm) for 4 h and then stained with calcein AM. Stained cells were added 96-well plates coated with fibronectin for a 4 h incubation. Non-adherent cells were removed and fluorescence was measured. (**e**) MM cell lines were incubated with idelalisib (1 μm), CZC24832 (1 μm) and duvelisib (1 μm) for 4 h and subsequently treated with calcein AM. Cells were then placed in the upper well of a 5 μm transwell plate. The lower chamber contained 30 μl of serum-free media or serum-free media supplemented with SDF1 (100 ng/ml) for 4 h and MM cell migration was then assessed using fluorescent plate reader.

The interaction between the myeloma cells and their microenvironment in the bone marrow is central in the regulation of tumour survival and progression. Surface adhesion molecules on MM cells have been shown to mediate cellular adhesion molecule drug resistance.^23^ We investigated the roles of PI3Kδ and PI3Kγ on MM cell adhesion to primary BMSCs to determine if suppression of the PI3K pathway could influence the engagement of cell surface integrins. MM cells were cultured with BMSCs at a ratio of 4:1 and cell cultures were incubated with idelalisib (PI3Kδ), CZC24832 (PI3Kγ) and duvelisib (PI3Kδγ all 1 μm) for 2 h before calcein AM fluorescence-based adhesion assays were used. Duvelisib, but not idelalisib or CZC24831, inhibited MM cell lines and primary MM cell adhesion to BMSC (Figures 4a and b). This data suggest that both isoforms function in the regulation of MM adhesion to BMSC through a combinatorial mechanism.

Inhibiting MM adhesion to BMSCs via the VLA4-VCAM/FN interaction is associated with increased tumour sensitivity to chemotherapy.^24^ We next examined if either PI3Kδ or PI3Kγ KD in MM cells could inhibit MM adhesion to FN. Figure 4c shows that PI3Kγ KD alone or in combination with PI3Kδ KD inhibits MM adhesion to FN. PI3Kδ KD had no effect on MM cell adhesion to FN. Next we wanted to determine if idelalisib (PI3Kδ), CZC24832 (PI3Kγ) and duvelisib could inhibit VLA4-VCAM/FN interactions. At the 1 μm dose used MM adherence to FN-coated plates was only inhibited by duvelisib (Figure 4d).

PI3K has been shown to regulate migration of immune cells including mast cells and neutrophils.^25, 26^ We hypothesised that inhibition of PI3Kδ and/or PI3Kγ subunits would inhibit MM migration towards SDF1 containing media. Figure 4e shows that MM cells migrate towards SDF1 supplemented media. Duvelisib (1 μμ) inhibited MM cell migration, however, this effect is not inhibited by either CZC24832 or idelalisib at similar drug concentrations.

### Together PI3Kδ/γ regulates IL-6-induced AKT activation in MM

**Figure 5:**
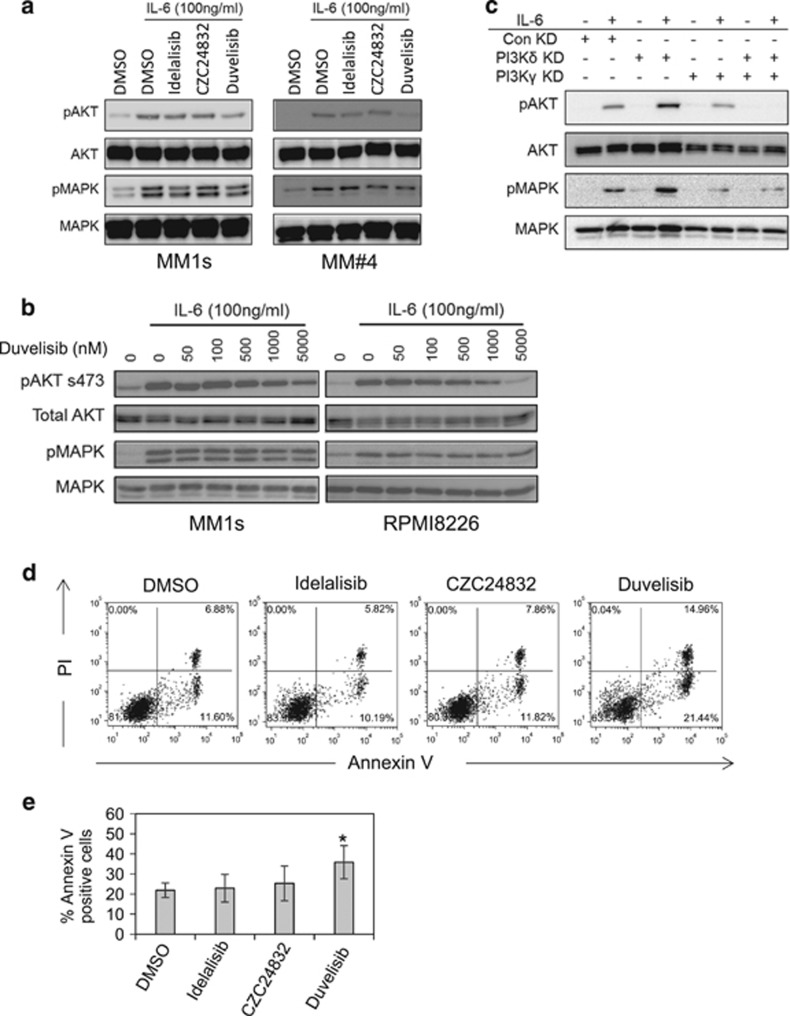
PI3Kδ/γ regulate IL-6-induced AKT activation in MM. (**a**) MM1s and primary MM were incubated with idelalisib (1 μm), CZC24832 (1 μm) and duvelisib (1 μm) for 4 h and then stimulated with IL-6 for 15 min before protein extraction. Samples were then analysed for phospho-AKT and phospho-MAPK response using Western blotting. Blots were re-probed to confirm sample loading. (**b**) MM1s and RPMI8226 cells were incubated with increasing doses of duvelisib for 6 h after which they were stimulated with and without 100 ng/ml IL-6 for 15 min. Protein was extracted and analysed for phospho-AKT and phospho-MAPK via Western blotting. Total AKT and MAPK were used as loading controls. (**c**) MM1s cells were transduced with lentivirus targeted to PI3Kδ and PI3Kγ or control shRNA for 72 h and then treated with IL-6 for 15 min. Protein was extracted and analysed for phospho-AKT and phospho-MAPK via Western blotting. Total AKT and MAPK were used as loading controls. (**d**, **e**) MM primary cells were incubated with BMSC for 24 h and then treated with idelalisib (1 μm), CZC24832 (1 μm) and duvelisib (1 μm) for 24 h, then assessed for apoptosis by PI/Annexin V staining.

IL-6 has been identified as a key cytokine supporting growth and proliferation of MM, as well as being shown to induce PI3K/AKT signalling.^27^ We therefore examined the effects of PI3Kδ and PI3Kγ inhibition on IL-6-induced activation of AKT and MAPK pathways. Figure 5a shows that duvelisib, but not CZC24832 and idelalisib, inhibits IL-6-induced AKT phosphorylation in serum-starved MM1s and primary MM samples. Figure 5b shows that duvelisib-induced inhibition is dose-dependent. To confirm these results we tested IL-6-induced activation of AKT in PI3Kδ KD, PI3Kγ KD and combined PI3Kδγ KD MM1s cells. Figure 5c shows that isolated PI3Kδ KD or isolated PI3Kγ KD failed to block IL-6-induced AKT activation; however combined PI3Kδγ KD completely blocks IL-6-induced AKT activation.

### BMSCs do not protect MM cells from PI3Kδ/γ inhibition

It has been shown that BMSC can protect MM cells from chemotherapy-induced apoptosis,^28^ so we decided to assess the efficacy of duvelisib in MM under BMSC co-culture conditions. Accordingly, we cultured primary MM cells with BMSC (ratio 4:1) in the presence or absence of duvelisib, CZC24832 or idelalisib (all 1 μm). Duvelisib induced apoptosis in primary MM cells (Figures 5d and e). These findings show that inhibition of PI3Kδ/γ with duvelisib induces apoptosis in MM cells even when co-cultured with BMSCs.

### PI3Kδ/γ inhibition significantly reduces engraftment and tumour burden *in vivo*

**Figure 6:**
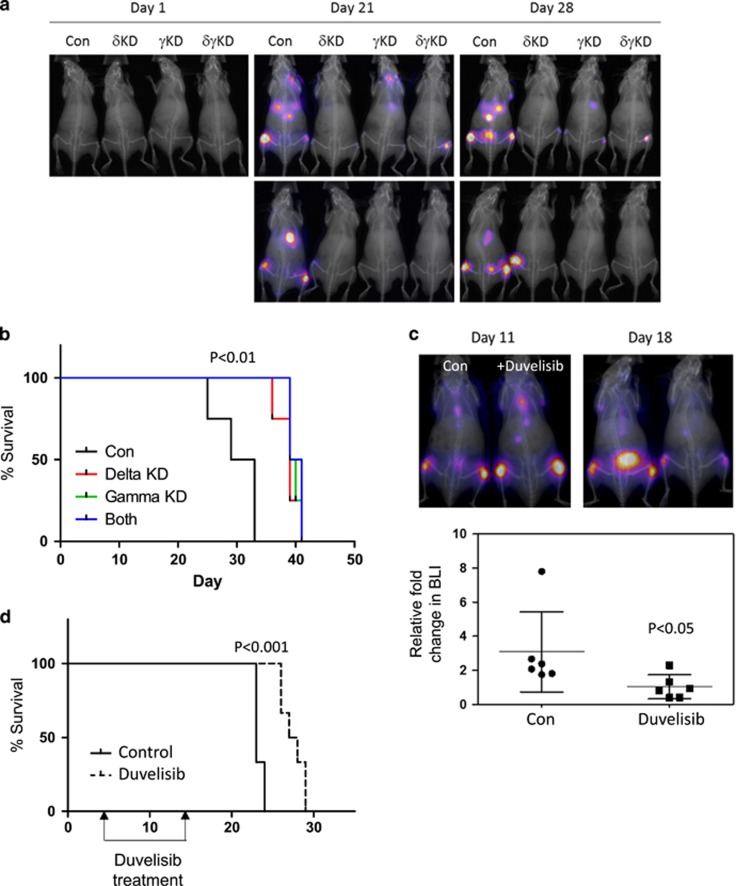
Inhibition of PI3Kδ/γ *in vivo* leads to reduced engraftment and increased survival. (**a**, **b**) U266-luc were transduced with lentivirus targeted to PI3Kδ and PI3Kγ or PI3Kδ/γ or control shRNA for 96 h. About 0.5 × 10^6^ cells lentiviral transduced U266-luc were subsequently were i.v. injected into NSG mice via the tail vein. (**a**) Bioluminescence (BLI) was used to track the U266-luc cells *in vivo*. (**b**) Survival of the NSG mice is represented by a Kaplan–Meier plot. (**c**, **d**) About 0.5x10^6^ cells were i.v. injected into NSG mice. At day 5 after i.v. injection mice were treated i.p. with IP1-145 (15 mg/kg per day) or vehicle control. (**c**) BLI was used to track the U266-luc cells *in vivo*. (**d**) Survival of the NSG mice is represented by a Kaplan–Meier plot.

In order to track MM disease progression *in vivo* we transduced U266 cells with a luciferase construct, which is detectable by bioluminescence in live animals. U266-luc cells were then transduced via lentiviral knockdown targeting of PI3Kδ and PI3Kγ. NSG mice (6–8 weeks) were injected with 0.5 × 10^6^-modified U266-luc cells from control, PI3Kδ, PI3Kγ or combined PI3Kδ/γ KD. At 21 days post injection, we analysed engraftment of U266-Luc cells by bioluminescence. Reduced engraftment was observed in animals transplanted with PI3Kδ, PI3Kγ or PI3Kδ/γ KD U226-luc cells compared to control KD U226-luc cells (Figure 6a). Figure 6b shows that animals transplanted with PI3Kδ, PI3Kγ or PI3Kδγ KD cells all have increased survival compared to control KD NSG mice.

To examine the effect of duvelisib *in vivo*, we injected mice with 0.5 × 10^6^ U266-luc cells via the tail vein. Mice were treated daily for 10 days with duvelisib (15 mg/kg on days 5–14 following transplant). On day 18 after transplant (4 days after completion of the 10- day duvelisib treatment), duvelisib-treated animals had reduced tumour burden when compared to vehicle control treated animals, as measured by bioluminescence (Figure 6c). Furthermore, Kaplan–Meier analysis shows significant survival improvement in the duvelisib-treated group when compared to the control-treated group (Figure 6d).

## Discussion

The PI3K p110 α, β, δ and γ subunits have all been shown to be constitutively expressed in MM.^11^ In this study we investigated the role of the PI3Kγ catalytic subunit and the effects of inhibiting PI3Kγ alone or in combination with PI3Kδ inhibition. We were interested to consider the biological rationale for the potential use of a clinical availability of a PI3Kδ inhibitor (idelalisib) or a combined PI3Kδ/γ inhibitor (duvelisib) in patients with MM. Here we report that drug inhibition of the PI3Kγ subunit reduces MM proliferation and survival. Furthermore, combined drug inhibition of PI3Kγ and PI3Kδ appears more cytotoxic to MM cells than inhibition of either isoform alone, an observation that is supported by data from isoform-specific shRNA-targeted KD experiments. This data are consistent with our finding that combined KD of PI3Kδ and PI3Kγ is more effective at inhibiting AKT phosphorylation than inhibition of either isoform alone. Functionally we found that MM adhesion to BMSC is regulated by both PI3Kδ and PI3Kγ, but FN adhesion and SDF1-mediated migration appear to be exclusively PI3Kγ-mediated processes. Moreover and of potential clinical relevance, BMSCs do not protect MM cells from combined PI3Kδ/γ inhibition. *In vivo*-combined PI3Kδ/γ inhibition significantly reduces MM engraftment. Finally, *in vivo*-combined PI3Kδ/γ inhibitor treatment reduces tumour burden and improves survival in animals with established MM. Taken together our data suggest that PI3Kδ and PI3Kγ appear to have both distinct and overlapping functions within MM and that inhibition of both isoforms simultaneously may result in the more effective form of treatment than single isoform inhibition alone.

AKT activity in MM is associated with poor patient prognosis and resistance to currently available treatment.^1, 2^ It is therefore a logical strategy to inhibit upstream AKT activators, the primary of which is PI3K. Previously, the PI3Kδ inhibitor idelalisib has been shown to reduce MM cell proliferation, however its effect on primary MM tissue had not been investigated.^11^ Moreover, despite the efficacy of pan-PI3K inhibitors on MM cells, these compounds appear to be poorly tolerated *in vivo* due to their lack of specificity and non-MM cell effects.^29^ Therefore given the early-phase clinical trial results with the dual PI3Kδ/γ inhibitor duvelisib, which shows the drug to be well tolerated in patients with lymphoma,^20^ we assessed the feasibility of dual PI3Kδ/γ inhibition in MM. In *in vitro* experiments we selected concentrations of drugs known to be biologically active but also achievable *in vivo*.^21^ Adhesion of MM cells to the bone marrow has been shown to be of critical importance regarding cell adhesion-mediated drug resistance.^23, 30^ In this study, we show that MM cell adhesion to BMSCs is significantly affected by suppression of the PI3Kδ/γ catalytic subunits in experiments with both targeted shRNA knockdown and drug inhibition with duvelisib. Lentiviral knockdown demonstrated the specific roles of the PI3K subunits, with PI3Kγ shown to have a more significant role in myeloma cell adhesion and migration.

The PI3K pathway contributes to the regulation of many cellular functions, including survival and proliferation, by catalysing the phosphorylation of phosphoinositide lipids.^31^ Aberrant PI3K pathway activation has been associated with several cancer types^32, 33, 34^ including MM and other haematological malignancies.^11, 31, 35^ Here we showed via Western blotting that the activation of this pathway is further upregulated in cell lines and primary samples when stimulated with the cytokine IL-6. IL-6 secreted from the BMSCs in response to MM has been shown to play a key role in MM pathogenesis,^36^ acting as a growth factor for MM cells and promoting drug resistance in the bone marrow microenvironment.^37, 38^ In addition, we show that lentiviral targeting of both PI3Kδ/γ subunits inhibits IL-6-induced AKT activation, whereas singular δ and γ KD does not have a significant effect. We therefore hypothesise that targeting both PI3Kδ/γ would be the preferred strategy to suppress the PI3K pathway in MM cells. Furthermore, we provide evidence that this should remain the case even when MM is subject to bone marrow-induced protection.

Lentiviral KD of PI3Kδ or γ or δγ in combination *in vivo* resulted in a reduction of disease burden and subsequent extension of survival in all conditions in comparison to the control. Percentage engraftment was significantly reduced in PI3Kγ-KD compared to the control KD, which suggests that targeting the PI3Kγ subunit could be a mechanism of reducing engraftment or even mobilising the MM cells from the bone marrow into the peripheral blood. The idea that PI3Kγ coordinates migratory signals is not novel and thus fits with the idea that targeting this isoform may be important in flushing the MM cells out of the bone marrow on which their survival is dependent.

Finally, we showed that treatment of MM-engrafted NSG mice with duvelisib resulted in a significant increase in the survival of the animals despite allowing for an engraftment period and treatment time of only 10 days. Tumour burden was shown not only to slow, inferring a reduction in proliferation, but actually reduce suggesting *in vivo* anti-MM activity for the drug. We did not conduct experiments to establish a formal drug-dosing strategy although it is encouraging from a clinical perspective that in other forms of haemic malignancy daily continuous dosing of PI3K inhibitors appears to generally well tolerated by patients.^22^ In conclusion, the data reported here provide a molecular mechanistic rationale for the clinical evaluation of duvelisib (and other combined PI3K γ/δ inhibitors) in patients with MM.
